# Priority measures to prevent infections and maintain residents’ well-being during COVID-19 outbreaks in nursing homes: Consensus among staff and resident representatives determined in an online nominal group technique study

**DOI:** 10.1016/j.ijnsa.2023.100142

**Published:** 2023-07-13

**Authors:** Lisa S. van Tol, Hanneke J.A. Smaling, Sarah I.M. Janus, Monique A.A. Caljouw, Wilco P. Achterberg

**Affiliations:** a University Network for the Care sector Zuid-Holland, Leiden University Medical Center, Leiden, The Netherlands; b Department of Public Health and Primary Care, Leiden University Medical Center, Leiden, The Netherlands; c Department of General Practice & Elderly Care Medicine, University of Groningen, University Medical Center Groningen, Groningen, The Netherlands

**Keywords:** COVID-19, Nursing homes, Infection prevention, Well-being, Measures, Policy making, Decision making

## Abstract

**Background:**

COVID-19 infection prevention measures can negatively impact nursing home residents’ well-being. Society has been concerned about the imbalance between infection prevention and residents’ well-being, and about nursing home residents’ autonomy in COVID-19 policymaking.

**Objective:**

This study explores consensus among nursing home staff about which measures they found to be most important in contributing to preventing infections and to maintaining well-being of residents during COVID-19 outbreaks. In addition, this study explores the decision-making processes regarding COVID-19 measures and the involvement of residents or their representatives.

**Design:**

Mixed methods based on an online nominal group technique.

**Setting(s):**

Dutch nursing homes, June–November 2020.

**Participants:**

Managers, policy advisors, elderly care physicians, psychologists, a spiritual counselor, nurses, care assistants, and resident representatives (*N* = 35).

**Methods:**

Four panels from the viewpoint of infection prevention, and four panels from the viewpoint of well-being were performed with 3 to 7 participants per panel. Participants individually selected the measure they found most important, discussed these measures together in an online conversation, and rated the importance and urgency of these measures during COVID-19 outbreaks on a 5-point Likert scale. The measures that were rated as (very) important and (very) urgent by all members of that panel were defined as ‘prioritized in consensus’. Panels also discussed the decision-making process regarding COVID-19 measures and the involvement of residents or their representatives. These conversations were transcribed verbatim and thematically coded using an inductive approach.

**Results:**

The infection prevention panels prioritized isolation measures; testing measures; testing and isolation combinations; use of personal protective equipment around (suspected) infected residents; and preparation for outbreaks by COVID-19 outbreak teams. The well-being panels prioritized cohort isolation, testing combined with cohort isolation and with isolation in residents’ rooms, exceptions to visitor bans, maximum numbers of visitors, and registration and accompanying visitors to the residents’ rooms. Resident representatives and staff were dissatisfied with their reduced involvement in policy making during the first months of the COVID-19 pandemic, although they understood that decisions had to be made quickly.

**Conclusions:**

Staff and resident representatives should be involved in COVID-19 policy making. According to them, priority COVID-19 measures should include: cohort isolation, testing and isolation combinations, use of personal protective equipment, crisis management by COVID-19 outbreak teams, and nursing home visit regulations and instruction of visitors. Combining these measures may be a first step towards packages of COVID-19 measures that better balance infection prevention and maintaining residents’ well-being.

**Registration:**

N/A

**Tweetable abstract:**

Priority COVID-19 nursing home measures are isolation, testing, testing and isolation combinations, PPE use, preparations by outbreak teams, and visit regulations @wilcoachterberg

**What is already known**

COVID-19 infection prevention measures can negatively impact nursing home residents’ well-being.

Society has been concerned that nursing home residents’ autonomy has been overlooked in COVID-19 policymaking.

Society has been concerned about the imbalance between infection prevention and residents’ well-being. Nursing home staff, residents, and their families have been struggling to develop better balanced packages of COVID-19 measures.

Alt-text: Unlabelled box

**What this paper adds**

Resident representatives and staff were dissatisfied with their limited involvement in policy making during the first months of the COVID-19 pandemic, although they understood that decisions had to be made quickly.

Priority measures during COVID-19 outbreaks in nursing homes include cohort isolation, testing, testing and isolation combinations, use of personal protective equipment, crisis management by COVID-19 outbreak teams, and nursing home visits regulations and instruction of visitors.

There is overlap between priority measures for preventing infections and for maintaining residents’ well-being during COVID-19 outbreaks in nursing homes: cohort isolation and combinations of testing and isolation were prioritized from both viewpoints.

Alt-text: Unlabelled box

## 1 Background

Among nursing home residents, elevated risks of severe disease courses of COVID-19 and mortality were seen (Comas-Herrera and Marczak, 2020; European Centre for Disease Prevention and Control ECDC, 2021; ...Ayalon and Zisberg, 2020). By May 2020, 37 to 66% of all COVID-19-related deaths in European countries had occurred in nursing homes and other long-term care facilities (Comas-Herrera and Marczak, 2020; Team and Danis, 2020). Inherent to the setting of nursing homes is close contact between people. Residents are often dependent on care that is provided by staff to multiple residents (European Centre for Disease Prevention and Control ECDC, 2021; Ayalon and Zisberg, 2020), and residents often share spaces, facilities, or activities (Ayalon and Zisberg, 2020; European Centre for Disease Prevention and Control ECDC, 2021; Gardner and States, 2020). COVID-19 therefore spreads easily among residents.

To protect vulnerable nursing home residents from COVID-19, many local and (inter)national guidelines and restrictions were applied during the first months of 2020, such as isolation measures, visiting bans or regulations, other distancing measures, testing, and use of personal protective equipment (Van Tol and Smaling, 2022; Dutch, 2019, British Broadcasting Corporation (BBC), Ecdc (2021a, 2021b), Comas-Herrera A and Lorenz-Dant, 2020). Although these measures should prevent COVID-19 transmission, public concerns arose about the lack of attention for nursing home residents’ well-being during spring and summer (WHO, 2021, Chason, 2020). Research confirmed that social isolation and loneliness, depressive symptoms, grief, anxiety, and challenging behavior had increased among nursing home residents during the first year of the COVID-19 pandemic (Benzinger and Kuru, 2021, Leontjevas and Knippenberg, 2021, Van Der Roest and Prins, 2020, Low and Hinsliff-Smith, 2021).

Researchers have suggested that infection prevention measures have to be weighed carefully against their potential harms (Gordon and Goodman, 2020) and person-centered care (Dichter and Sander, 2020, Dykgraaf and Matenge, 2021, Dohmen and Van Den Eijnde, 2022) in order to maintain residents’ well-being. However, how these considerations are to be made in practice is not described (Gordon and Goodman, 2020, Dichter and Sander, 2020). Nursing home staff, residents, and residents’ family members have also been struggling to find packages of measures that provide a better balance between infection prevention and maintaining residents’ well-being (Leontjevas and Knippenberg, 2021, Gerritsen et al., 2022). Staff and residents’ family feared for residents’ safety and argued in favor of even stricter infection prevention measures, although they also found visitor restrictions difficult to cope with and campaigned for residents’ freedom of movement (Gordon and Goodman, 2020, Smaling and Tilburgs, 2022).

Another public concern was that nursing home residents’ autonomy has been overlooked in COVID-19 policymaking (Low and Hinsliff-Smith, 2021, Gordon and Spilsbury, 2022). It was argued that, especially due to their relatively short remaining life expectancy, many nursing home residents had died, or would die, under restrictive measures they had no say in Chu and Donato-Woodger (2020). However, the actual decision-making process in nursing homes and the involvement of residents regarding COVID-19 measures has hardly been studied.

In response to these two public concerns, the first aim of this study is to explore consensus among nursing home staff and resident representatives about measures they found to be most important in contributing to preventing infections, and to maintaining residents’ well-being during COVID-19 outbreaks in nursing homes. The second aim is to explore how the decision-making processes and the involvement of residents or their representatives regarding COVID-19 measures were organized and experienced in nursing homes.

## 2 Methods

### 2.1 Design

This study has a mixed-methods design using the nominal group technique (NGT) (Mcmillan and Kelly, 2014, Potter and Gordon, 2004, Gallagher and Hares, 1993). The NGT was slightly modified to an online procedure with a single rating phase. This consensus method has been used to identify COVID-19 measures that nursing home staff members and resident representatives give priority to. This study made use of participants and data from the MINUTES study (Van Tol and Smaling, 2021).

### 2.2 Participants

Long-term care organizations that participated in the MINUTES study (Van Tol and Smaling, 2021) were invited to participate. Since the focus of our study is on inpatient long-term care, these were all organizations with at least one nursing home or care home. An information letter was sent by email to the directors of these 41 long-term care organizations. Consent to study participation was received in reply to this email from 12 organizations. Next, the contact person of each participating organization distributed information letters to potential participants. Besides, we asked potential participants if they knew any colleagues who might be interested in receiving an information letter and considering participation in a next round of panels. When potential participants indicated interest in participating, we checked if there were not yet two participants from the same long-term care organization or with the same function registered for the next round of panels. Subsequently, we asked for their availability during the times of the scheduled online panel conversations and if they preferred to participate in a panel regarding infection prevention measures or about well-being measures.

The aim was to organize panels of 4 to 7 participants, to keep the online process manageable (Mcmillan and Kelly, 2014). To include diverse perspectives of stakeholders in nursing homes’ policy making, we aimed to compose panels that consisted of multidisciplinary nursing home staff from various long-term care organizations, including managers or policy advisors; practitioners such as physicians, psychologists, or physiotherapists; nurses or care assistants; and resident representatives.

### 2.3 Setting

Shortly before the start of this study, in May 2020, nursing homes gradually started to reopen for visitors (Sizoo and Monnier, 2020), and the Dutch ‘first wave’ of national excess mortality due to coronavirus infections was just over (CBS). However, nursing homes were still short of personal protective equipment (Marijnissen et al., 2021). Right at the beginning of the study period, in June 2020, tests became available for Dutch citizens, including nursing home residents, with symptoms of COVID-19 (CBS). From September 2020 until June 2021, the indistinguishable second and third waves of coronavirus infections were ongoing (CBS). By the end of the study period in November 2020, there were no personal protective equipment shortages anymore, but restrictive measures, e.g., to limit group sizes, had been reintroduced at the national level (Rijksinstituut voor Volksgezondheids en Milieu). Vaccines were not yet available. Rounds of panels were organized in June, July, August and November 2020.

### 2.4 Procedures

The NGT is a structured group procedure for idea generation, problem solving, and priority setting that encourages equal input from all participants (Gallagher and Hares, 1993). A modified NGT was employed to define levels of agreement about priority COVID-19 measures among nursing home staff. Due to national COVID-19 restrictions, the study had to take place online. To limit the burden on the participants in a time of crisis, and because our aim was not to enforce consensus, but only to explore the existing level of consensus in this early stage of the pandemic, there was no secondary ranking or re-ranking, which is often the last phase of the NGT (Mcmillan and Kelly, 2014, Gallagher and Hares, 1993). Multiple rounds of NGT panels from each viewpoint were conducted, until data saturation was reached in the topics of measures selected to be most important. The phases of the NGT are presented in Table 1.

**Table 1:** Procedures nominal group technique.

Phase	Format	Content/structure
1. Idea generation	Form by email	Open questions: 1. “What measure, taken in the nursing homes organizations you are affiliated with, do you think is most important to implement during a COVID-19 outbreak in nursing home organizations [to prevent infections /to maintain residents’ well-being]?” 2. “Why this measure?” 3. “From whose perspective(s) was this measure decided? Was there participation/ consultation/ questioning of client(council)s?” 4. “Does this differ from the way policy is made in the normal situation? What do you think about that?” Attachment: list of textual units from the last four weeks of MINUTES data that described measures.
2. Panel conversation	Online via Zoom	Conversation part 1: measures (phase 1 questions 1 and 2) - step 1. Round robin: participants one by one explain their answers to open questions 1 and 2 - step 2. Discussion: participants ask each other clarifying questions and discuss their ideas Break Conversation part 2: decision making (phase 2 questions 3 and 4) - step 1. Round robin: participants one by one explain their answers to open questions 3 and 4 - step 2. Clarification and discussion
3. Rating	Form by email	5-point Likert scale questions: 1. “This measure is important for [infection prevention/the well-being of residents and their family] during COVID-19 outbreaks in the nursing home” 2. “Introducing this measure at the start of a COVID-19 outbreak in the nursing home is urgent, and cannot wait weeks” Comment field

Phase 1, the idea generation phase, consisted of a questionnaire via email one week before the panel meeting. The questionnaire contained questions about the most important measure during COVID-19 outbreaks from the viewpoint of either infection prevention or well-being, and about the decision-making process (Table 1). To activate participants’ thoughts, a list of textual units from the MINUTES data (Van Tol and Smaling, 2021) of the previous four weeks was attached to the questionnaire. As an example, **Supplement I** presents the attachment for the first panel about infection prevention measures.

In phase 2, the 1.5 to 2-hour panel conversations were conducted online via Zoom (Zoom Video Communications 2022) and were guided by a facilitator, an assistant facilitator who also served as technical assistant before and during the conversation, and one person taking minutes (female researchers JG, LT, and SJ in alternating roles). The information letter contained technical instructions regarding Zoom, and shortly before and during the meeting, the assistant facilitator was available to provide participants with technical support. The conversations followed usual NGT steps: a round robin and a discussion. With a 10-minute break in between, these steps were performed for conversation part 1 regarding the selected measures and for part 2 regarding the decision-making process. The panel conversations were audio recorded.

Phase 3, rating of the measures discussed, was conducted by email one week after the panel conversation. Participants received a form with descriptions of the measures, including arguments and nuances that were mentioned during the conversation. They were asked to rate the importance and the urgency of each measure on a 5-point Likert scale. In addition, the form served as a member check (Varpio L et al., 2016). Descriptions of the measures were adapted if participants’ comments indicated an inaccurate interpretation of the conversation.

### 2.5 Data analysis

The discussion elements of the panel conversations about important COVID-19 measures were transcribed verbatim. Between phases 2 and 3, the transcripts of conversation part 1, regarding COVID-19 measures, were analyzed using content analysis (Potter and Gordon, 2004, Vaismoradi and Turunen, 2013) and converted into the measure descriptions on the rating forms (LT, checked by JG and SJ). After phase 3, the rated measures were classified under the eight main topics that were identified in the MINUTES study as topics of discussion in COVID-19 outbreak teams: crisis management, isolation of residents, personal protective equipment and hygiene, staff, residents’ well-being, visitor policies, testing, and vaccination (Van Tol and Smaling, 2021) (LT, checked by HS and MC). This classification facilitated comparison between the measures prioritized by the well-being panel and by the infection prevention panel. Measures were considered to be prioritized in consensus if they were ranked as (very) important (4 or 5 points) and (very) urgent (4 or 5 points) by all panel members. The transcripts of part 2 of the panel conversations, regarding decision making, were inductively thematically analyzed (LT, reviewed and discussed with HS) (Vaismoradi and Turunen, 2013).

### 2.6 Ethics

The study was exempt from the Medical Research Involving Human Subjects Act by the Medical Ethics Committee Leiden The Hague Delft (protocol number N20.093). The directors of the long-term care organizations and all panel participants provided written informed consent. The study was announced on the Long-Term Care response to COVID-19 website on March 10, 2021 (LTCcovid.org).

## 3 Results

### 3.1 Participants

Four rounds of panels on infection prevention and on well-being were performed. After for rounds, data saturation was reached in all topics except crisis management. This topic was assumed to indicated that by November, nursing homes no longer experienced the pandemic as a crisis but reflected on and prepared for future crisis situations. In total, 19 participants participated in the infection prevention panels, and 16 in the well-being panels. Each panel consisted of 3 to 6 participants. Their median ages were 55 (range 30–73) and 50 (range 21–73) years, 68% and 56% were female, and their median years of experience in their positions were 8.0 (3.0–24.0) and 5.5 (1.6–7.8) years, respectively. Participants included care assistants, elderly care physicians, managers, nurses, policy advisors, psychologists, resident representatives, and a spiritual counselor (Table 2). There were no panels with more than one pair of participants from the same function or more than one pair of participants from the same long-term care organization. The resident representatives were all members of nursing home client councils and current or former family caregivers of nursing home residents.

**Table 2:** Participant characteristics.

	Infection prevention panels (*n* = 19)	Maintaining well-being panels (*n* = 16)
**Sex**		
Female	68.4% (*n* = 13)	56.3% (*n* = 9)
**Age** (Median (range))	55 (30–73)	50 (21–73)
**Function**		
Care assistant*	5.3% (*n* = 1)	6.3% (*n* = 1)
Elderly care physician	10.5% (*n* = 2)	N/A
Manager⁎⁎	21.1% (*n* = 4)	31.3% (*n* = 5)
Nurse	15.8% (*n* = 3)	18.8% (*n* = 3)
Policy advisor	26.3% (*n* = 5)	6.3% (*n* = 1)
Psychologist	N/A	12.5% (*n* = 2)
Resident representative	15.8% (*n* = 3)	25.0% (*n* = 4)
Spiritual counselor	5.3% (*n* = 1)	N/A
**Years in position** (Median (IQR))	8.0 (3.0–24.0)	5.5 (1.6–7.8)

⁎ care assistants were a ‘carer’ and an ‘activity supervisor’.

⁎⁎ one manager was also chair of the pre-pandemic infection prevention committee.

### 3.2 Infection prevention measures

The 19 participants in the panels about infection prevention selected and discussed 16 different measures. These 16 measures were on the topics of isolation, testing, testing and isolation combinations, personal protective equipment, hygiene, visiting measures, and crisis management. Of these 16 measures, 9 were prioritized as being (very) important and (very) urgent in nursing homes during COVID-19 outbreaks. These 9 priorities are described below (Table 3).

**Table 3:** Prioritization of measures preventing infections during COVID-19 outbreaks in nursing homes (nominal group technique phase 3).

Measure	Panel (month)	Importance scores						Urgency scores					
		5	4	3	2	1	*missing*	5	4	3	2	1	*missing*
Isolation													
Isolate wards with infected residents as cohorts and restrict entry to limited number of staff members with PPE*	**Jun**		**2**				***2***	**2**					***2***
**Transfer infected residents who cannot be isolated on own ward to a COVID-19 unit**	**Jul**	**2**	**3**					**2**	**3**				
**Isolate residents with (suspected) COVID-19 who are able to comply with isolation measures in a single room**	**Aug**	**3**	**1**				***1***	**3**	**1**				***1***
Transfer residents with (suspected) COVID-19 not able to comply with isolation measures to a COVID-19 unit	Aug		2	2			*1*		3	1			*1*
Tailor isolation measures for specific locations, wards, and for residents by their physician	Aug	2		2			*1*	1	2	1			*1*
Testing													
**Test residents and staff with (minor) symptoms and isolate them in the case of a positive result**	**Jul**	**4**	**1**					**3**	**2**				
**Test staff and residents within the care organization itself if national testing resources are scarce**	**Nov**	**2**	**3**					**2**	**2**				***1***
Testing and isolation combinations													
**Test and transfer residents suspected of COVID-19 to a COVID-19 unit with experienced staff**	**Jun**	**1**	**1**				***2***	**1**	**1**				***2***
**Test and isolate residents who are (possibly) infected until a negative test result is obtained**	**Nov**	**4**	**1**					**4**	**1**				
PPE*													
Use personal PPE*around (suspected) infected residents	**Jul**	**4**	**1**					**4**	**1**				
Use of mouth-nose masks as a precaution by all staff members who enter wards with residents	Nov	3	1	1				4		1			
Crisis management													
**Preparation for outbreaks by a COVID-19 outbreak team**	**Nov**	**3**	**2**					**3**	**2**				
Hygiene													
Raise staff's awareness of general hygiene, which includes work clothes, PPE*, jewelry, and hand hygiene	Jun	2					*2*	1		1			*2*
Keep hygiene and infection prevention policies up to date and share the content with staff	Jul	3	1	1				2	1	2			
Visiting policies													
Ban all visitors other than staff, including residents’ families, volunteers, and suppliers	Jun	1		1			*2*	1			1		*2*
Decentralize policy making about strict visiting instructions and regulating the flow of visitors	Aug	2	1	1			*1*	2	2				*1*

Note: measures in bold are prioritized in consensus.

⁎ PPE: personal protective equipment.

Of the 9 prioritized measures, 3 concerned the topic of isolation. These isolation measures varied from cohort isolation of wards with infected residents and entry restricted to a limited number of staff members with personal protective equipment (June); transfer of infected residents who cannot be isolated on their ward to an organizational or regional COVID-19 unit (July); and isolation of residents with (suspected) COVID-19 who are cognitively able to comply with this measure in their single room (August). Panel members argued that transfer and isolation in a COVID-19 unit would stimulate a regionally uniform policy, centralize experiential knowledge on caring for COVID-19 patients, and centralize usage and therefore save personal protective equipment. Isolation in single rooms would prevent transfer of residents to a COVID-19 unit with staff who are not familiar with their normal behavior. Cohort isolation was argued to prevent transfer so residents can stay in their familiar environment as well as prevent isolation in single rooms so residents retain some freedom of movement.

Four measures were prioritized in the topics of testing (*n* = 2), and testing and isolation combinations (*n* = 2). The testing measures were, first, immediate testing of residents and staff in the event of (minor) symptoms, and isolation in case of a positive test result (July). Second, testing of staff and residents should be performed within the care organization itself if national testing resources are scarce or processes are slow (November). The prioritized combinations of testing and isolation were testing and transferring residents suspected of COVID-19 to a COVID-19 unit with experienced staff, especially when test results are not quickly available (June); and, in nursing homes where the building structure makes it difficult to prevent transmission of the virus, testing and isolating residents who are (possibly) infected until a negative test result is obtained (November). Both panels argued that testing can shorten the period during which isolation measures are needed.

Measures prioritized in other topics (*n* = 2) were, first, the use of personal protective equipment around (suspected) infected residents (July). This measure was argued to reduce unrest among staff. Second, having an organizational COVID-19 outbreak teams to prepare for outbreaks was prioritized (November). This was aimed at reducing fear and work overload of staff.

### 3.3 Measures to maintain residents’ well-being

The 16 participants in the well-being panels selected and discussed 14 different measures. These 14 measures were on the topics of visitor policies, isolation, testing and isolation combinations, crisis management, and activities for residents. Of these 14 measures, 8 were prioritized as being (very) important and (very) urgent to maintain the well-being of nursing home residents during COVID-19 outbreaks by all panel members. These 8 priorities are described below (Table 4).

**Table 4:** Prioritization of measures maintaining residents’ well-being during COVID-19 outbreaks in nursing homes (nominal group technique phase 3).

**Measure**	**Panel (month)**	**Importance scores**						**Urgency scores**											
		5	4	3	2	1	*missing*	5		4		3		2		1		*missing*	
Visiting policies																			
**Lift visitor bans in the case of terminal residents and any other distressing cases**	**Jun**	**4**							**4**										
Allow residents in the dying phase to receive 2 or 3 visitors daily with PPE*	**Jul**	**2**	**1**						**2**		**1**								
**Register visits and accompany visitors to the residents’ rooms**	**Aug**	**1**	**3**						**3**		**1**								
**Do not permit visitors to visit residents or wards that have tested positive**	**Aug**	**2**	**2**						**4**										
**Limit the daily number of visitors to 2 or 3 of the residents’ choice during COVID-19 outbreaks**	**Nov**	**3**	**2**						**3**		**2**								
Ban visitors or allow only regulated visiting, tailored to residents’ wishes	Jun	1	1	2					1				1		2				
Facilitate ‘visiting windows’ where family and residents can see and (video) call each other	Jun	1	2	1					2		1		1						
Isolation																			
**Isolate ward with residents who are (suspected of) having COVID-19 as a cohort**	**Jul**	**2**	**1**						**2**		**1**								
**Tailor cohort isolation to the smallest possible units or wards around a COVID-19 outbreak**	**Aug**	**3**	**1**						**4**										
Cohort wards and compartment staff to cohorts to allow living room activities to continue within cohorts	Jun	1	3						1		2		1						
Testing and isolation combination																			
**Test and isolate residents with suspected COVID-19 in their rooms; and in case of positive test results, isolation the entire ward as a cohort**	**Jul**	**3**							**3**										
Crisis management																			
Appoint a crisis manager or crisis team	Nov	1	2	1	1				2		1		2						
Organize evaluation conversations on COVID-19 measures	Nov	3	2						2				3						
Well-being activities																			
Continue indoor and outdoor activities for residents from the same wards	Jul	2	1						1		1		1						

Note: measures in bold are prioritized in consensus.

⁎ PPE: personal protective equipment.

Of the 8 prioritized measures, 5 concerned the topic of visitor policies. One panel prioritized not permitting visitors to visit residents or wards with positive test results, but allowing visitors who wear mouth masks to visit residents who are suspected of having COVID-19 (August). Two panels prioritized exceptions to visitor bans for terminal residents and other distressing cases (June) and for residents in the phase of dying (July). This last panel wanted to allow a maximum of 2 or 3 visitors daily with personal protective equipment. Two panels furthermore prioritized registration of visits and accompanying visitors to the residents’ rooms (August), a maximum of 2 or 3 visitors of the residents’ choice per day per resident during COVID-19 outbreaks, and additional clear restrictions such as location of visits, mandatory mouth masks, and registration of visits (November). These panel members argued that regulation of visits has less impact on residents’ and their family members’ daily lives and intimacy and on the workload for staff, than visitor bans. In addition, all visiting measures other than visitor bans were argued to be important to maintain residents’ autonomy or privacy.

The other three prioritized measures concerned the topics of isolation and testing and isolation combination. These are overlapping with measures prioritized by the panels about infection prevention. The figure in **Supplement II** illustrates this overlap. Cohort isolation should be applied to wards with (suspected) infected residents (July), and tailored to the smallest possible units or wards around a COVID-19 outbreak (August). Participants argued that cohort isolation to the smallest possible unit or ward would keep the impact on residents, their family, and staff to a minimum (August), and keep other wards within the building accessible for visitors (July). In addition, testing and isolating residents with suspected COVID-19 in their room (droplet isolation) was prioritized. A positive test result would require transition from room isolation to cohort isolation of the ward (July).

### 3.4 Decision making

Most participants were aware of how decisions regarding COVID-19 measures were made within their organization. They explained that the COVID-19 outbreak teams made decisions more quickly and more top-down than usual, often implementing national COVID-19 policies (**Supplement III: quote 1 (Q1))**. However, they did not always know of whom the outbreak teams consisted. Resident representatives were often not involved at all, and were merely informed of COVID-19 policies. Often they only remained involved in the topic of visitor policies **(Q2)**. Barriers to their involvement in the decision-making included not being able to get together on location, to meet with staff, and COVID-19 outbreak teams meeting ad hoc (**Q3**). One panel discussed the possibility of including a resident representative in a COVID-19 outbreak team, but this panel's participants were divided about this idea.

Several staff members indicated feeling they were not heard by the COVID-19 outbreak team (**Q4).** Some staff members explained that they were informed of decisions about COVID-19 measures, but that their questions, or questions they received from residents, family members, and other staff remained unanswered.

While resident representatives and staff expressed dissatisfaction and irritation about their decreased involvement (**Q5**), they also indicated understanding the accelerated decision-making process during the first months of the pandemic (**Q6**). The panels of August and November indicated that staff and client councils were increasingly involved in COVID-19 policy making, and that staff was given more freedom in applying policies (**Q7**).

According to one participant, the decision making process did not differ much from before the pandemic, because it was usual that deviations from ‘normal’ care were discussed with the manager or physician who were now part of the COVID-19 outbreak team.

## 4 Discussion

This study examined a set of COVID-19 measures nursing home staff and resident representatives deemed most important and urgent to prevent infections and maintain residents’ well-being during COVID-19 outbreaks in nursing homes. They prioritized cohort isolation and testing and isolation combinations to both prevent infections and maintain residents’ well-being. In addition, to prevent infections, more isolation measures, testing measures, use of personal protective equipment around (suspected) infected residents, and preparation for outbreaks by COVID-19 outbreak teams were prioritized; and to maintain residents’ well-being, exceptions to visitor bans and several visitor policies were prioritized. These measures combined may be a first step towards packages of COVID-19 measures that better balance infection prevention and maintaining well-being. Resident representatives and staff were dissatisfied with their reduced involvement in policy making during the COVID-19 pandemic, although they understood that decisions had to be made quickly. These prioritized sets of measures are a first step towards packages of COVID-19 measures that better balance infection prevention and maintaining residents’ well-being.

This is the first study to prioritize COVID-19 measures in terms of importance and urgency during outbreaks in nursing homes to both prevent infections and maintain residents’ well-being. In line with our findings, priority research areas regarding infection prevention that have been designated academic authors and specialist societies include testing and vaccination, and use of personal protective equipment; and also well-being priorities including consequences of COVID-19 for physical, cognitive and psychological health, and the impact of social distancing and lock-down policies (Richardson and Carroll, 2020). Besides, two literature reviews described COVID-19 best practices and potentially effective measures that are mainly in the topics that our study rated to be important, including (cohort) isolation, testing, combining testing and isolation, use of personal protective equipment, hygiene reinforcement, visiting policies, and crisis management (Dykgraaf and Matenge, 2021, Martinez-Paya and Carrillo, 2022). In addition to our results, these reviews also described ventilation, digital health applications such as real-time outbreak monitoring, workforce management (Dykgraaf and Matenge, 2021), and person-centered care (Martinez-Paya and Carrillo, 2022) to be important. Although person-centered care may refer to residents’ well-being, these reviews included mostly studies that were only focused on infection prevention, Moreover, many of the studies included in these reviews did not include the perspective of nursing home staff, residents, or resident representatives.

In our study testing and isolation combinations were also prioritized by the well-being panels, although intuitively these may be only infection prevention measures. A plausible explanation is that participants were inclined to select infection prevention measures because they experienced mainly this type of measures in practice during the first months of the pandemic. The well-being measures reflect that relaxation of infection prevention measures benefits residents’ well-being. Besides, this might also be explained by the CDC definition of well-being, which besides mental, social, and autonomy components, also includes a physical component (Well-Being Concepts)*.* Nursing home staff might indeed have regarded infection prevention as part of maintaining physical health and an integral part of well-being. Future research could reveal if the focus regarding well-being has changed after the acute crisis passed.

Our study showed that the autonomy of nursing home residents should be maintained during COVID-19 outbreaks. According to literature, the norm is to involve them or their representatives in care decisions and to deliver person-centered care (Leontjevas and Knippenberg, 2021, Roach and Zwiers, 2021). Dutch care organizations are obliged to have a client council that is informed about, can give advice, and can consent to organizational policy decisions (Well-Being Concepts 2019). Despite these norms and obligations, internationally involvement of nursing home residents in care and policy decisions remains difficult (Lynch and Ryan, 2022). With regard to COVID-19 measures, researchers have also advised tailor-made measures and the involvement of residents, family, and staff (Van Tol and Smaling, 2022; Dutch, 2019, Van Der Roest and Prins, 2020, Dichter and Sander, 2020, Dohmen and Van Den Eijnde, 2022). However, our study shows that resident representatives and nursing home staff were less involved in policy making during the first months of the COVID-19 pandemic than before. In fact, according to another Dutch study opinions of staff and resident representatives about who was responsible for policy making even became divided (Visser et al., 2022). This may be due to hierarchical (Dykgraaf and Matenge, 2021) centralized quick policy making during the first months of the pandemic (Gordon and Spilsbury, 2022). However, our results showed that by November 2020 there was more room for involvement in the decision-making processes within nursing homes. It has been suggested that, ultimately, these first months of the COVID-19 pandemic may have been a catalyst for a bottom-up urging to better embed involvement in the nursing home sector (Chu and Donato-Woodger, 2020).

### 4.1 Strengths

A first strength of this study is the bottom-up perspective. Our participants represented disciplines that ideally take part in nursing home policy making, ranging from nursing home managers to care assistants, and resident representatives. It is crucial that they support the policy decisions made (Chu and Donato-Woodger, 2020, Centers for Disease Control and Prevention CDC, 2021). Second, this study is unique in its prioritization of COVID-19 measures. We made use of a modified NGT to quickly generate results that are easily translatable to nursing home policies and practice. Many different COVID-19 measures have been recommended and implemented, and it has been difficult to select a balanced package of COVID-19 measures. The NGT has much been used to explore priorities and for policy development about other healthcare issues (Mcmillan and Kelly, 2014, Potter and Gordon, 2004). Third, performing this NGT study online made it possible to collect data about COVID-19 measures during the national COVID-19 restrictions. In addition, by performing the study online, participants from across the country were able to participate without traveling (Willemsen and Aardoom, 2022, Mason and Ling, 2021).

### 4.2 Limitations

Limitations of this study were, first, the small numbers of participants per panel. Still, after four rounds of panels, data saturation was reached. Second, this study was performed during the first year of the COVID-19 pandemic, when vaccines were not available yet. Recent literature stresses the importance of vaccination. Vaccination might have changed the urgency of other measures to prevent infections and maintain well-being in nursing homes. Future research should explore this renewed balance. Third, effectiveness of measures in preventing infections or maintaining well-being remains unknown. In the first phase of the COVID-19 pandemic, effectiveness studies would have been too time consuming, and it would have been ethically irresponsible to withhold potentially effective measures from non-vaccinated NH. In this study, participants’ arguments in favor of the prioritized measures included perceived effectiveness, but also feasibility in practice and ethics. Thus, the measures prioritized should be the first focus in future effectiveness studies. Fourth, although formal resident representatives from client councils participated in this study, no nursing home residents participated. All of the client representatives willing to participate were current or former family caregivers. They may have been more familiar with digital communication than most residents. As described in the study background, family members feared for residents’ safety and may therefore have selected more infection prevention measures to maintain well-being than residents themselves would do. Future research should also explore which measures have priority from the perspective of residents themselves.

## 5 Conclusions

In conclusion, this study has laid bare that long-term care organizations and the Dutch government were not well-prepared and had no policy in place for a highly infectious disease epidemic in the sector. However, Dutch long-term care organizations were flexible and reactive. During future times of crisis when decisions are made more quickly than usual, staff and residents or their representatives should be better informed about the decision making processes, more involved in decisions, and have more opportunities to ask questions than they had during the first months of the COVID-19 pandemic. Long-term care organizations may draw up a crisis communication and involvement protocol that includes sharing weekly updates with staff. In addition, staff should be given room to tailor-make COVID-19 measures. Priority measures during COVID-19 outbreaks in nursing homes to prevent infections and to maintain residents’ well-being partly overlap, and include cohort isolation and other types of isolation, testing measures, testing and isolation combinations, use of personal protective equipment around (suspected) infected residents, crisis management by COVID-19 outbreak teams, and regulation of visits and instructing visitors. Despite public commotion regarding the perceived imbalance between infection prevention and maintaining well-being during the first months of the pandemic, the measures prioritized to maintain residents’ well-being seem to mostly reflect gradual relaxation of infection prevention measures. This may be caused by the fact that mainly this type of measures were taken and experienced by the study participants. Future research may reveal whether after the acute crisis situation other topics also have gained priority to maintain residents’ well-being during infectious disease outbreaks. These prioritized sets of measures are a first step towards packages of COVID-19 measures that can overcome the perceived imbalance between infection prevention and maintaining residents’ well-being. The next step should be to let nursing home staff and residents jointly weigh the prioritized measures. For now, the study results can be translated to nursing home policies and can be used to provide focus to crisis management in future outbreaks of highly infectious or unknown respiratory viral diseases in nursing homes. When doing so, with each measure the effect on residents’ well-being should be considered.

## Funding sources

This research was funded by the Dutch ministry of Health, Welfare and Sport, grant number 331873*.*

## Declaration of Competing Interest

None.

## Appendix Supplementary materials

Image, application 1

Image, application 2

Image, application 3
