# Supplementary material for: Priority measures to prevent infections and maintain residents’ well-being during COVID-19 outbreaks in nursing homes: Consensus among staff and resident representatives determined in an online nominal group technique study
Source: Int J Nurs Stud Adv. 2023 Jul 13;5:100142. doi: 10.1016/j.ijnsa.2023.100142 (PMC11080460; doi:10.1016/j.ijnsa.2023.100142)
Supplement: Supplementary file 1 [file mmc1.docx]

**Supplement I: list of textual units from the MINUTES data - attachment to the idea generation questionnaire of June’s panel on infection prevention**

| **Segregation and isolation of clients** |
| --- |
| - Separate locations such as care hotels are set up as COVID centers |
| - Anteroom area available to isolate room and for donning personal protective equipment (PPE). |
| - Discharge procedure from COVID ward has been established. Definition no longer infectious: when client is symptom-free for 24 hours, at least 14 days after the start of the symptoms, and when client has had droplet isolation for 7 days after the 24 hours symptoms-free. |
| - Restaurant may reopen 14 days after positive test of last client. |
| - Care agreement stops when a client goes home with a family caregiver. |
| - From multiple- to single-bed rooms on [ward X]. |
| - Signage (for one-way routes) is made compulsory. Where too narrow for one direction, (expensive) traffic lights can be used. |
| - Local residents with physical therapy inside a location are only allowed in if there is a separate entrance and exit. |
| - Stop on internal transfers and new admissions. |
| - 2 infections on 1 ward considered as whole ward infected, admissions freeze. |
| - Chairs with upholstery and cabinets with knickknacks removed from Covid cohorts |
| - Locations with a terrace on the ground floor will be cordoned off with tape. |
| - Upon return after leaving location, client must be quarantined for 2 weeks. |
| - Rehabilitation wards of 7 locations become COVID-cohorts. |
| - Moving clients who have tested positive is avoided. |
| - Isolate suspected/infected client in own room. If not feasible (due to behavioural problems/client unable to follow instructions/serious aggression/inappropriate accommodation), client is transferred to cohort ward. |
| - Residents in individual isolation can be transferred to Covid-Cohort by mutual agreement. |
| - Negatively tested clients are also quarantined for 24 hours as a precaution. |
| - If a resident with an urge to wander and suspected corona-virus infection, the doctor decides how best to isolate the resident. In practice, this will involve confinement to the room or sedation. |
| - After three days symptom-free and at least 8 days after diagnosis by test, the isolation can be lifted. In case of doubt, a second test (at least 24 hours apart) can be performed. |
| - Admissions freeze infected location. |
| - New admissions who were not tested in hospital are tested prior to admission. |
| - PG (psychogeriatrische) cliënten met loopdrang zijn moeilijk instrueerbaar (moeilijk in quarantaine te houden) en worden daarom niet opgenomen. |
| - Housekeeping will not clean rooms of positive clients. This is done by the care workers themselves. |
| **New admissions** |
| - After a death, a bed in the nursing home can be released for a new resident. If it is a bed in a covid-cohort, only a new resident with (suspected) covid can be admitted. |
| - When new clients move in, they do not have to be quarantined for 14 days, unless they have symptoms. |
| - Upon admission, 2 weeks isolation policy (from hospital, care institution or home situation), temperature taken 2x a day, resident stays in own room. |
| - Process of moving in clients is discussed in broad outline and adjusted where necessary (e.g., guided tour via video calls, professional removal company moving personal belongings). |
| **Hygiene** |
| - Before bedding is taken away to be washed by an external party, the bedding must first be packed airtight for 24 hours. |
| - Place hand disinfection columns for visitors. |
| - In case of infection, we clean contacts with alcohol/disinfectant. Other areas with microfibre and water. |
| - Cleaners pay extra attention to contact points like (door)handles and handrails. |
| - Keyboards, pagers, etc. may be disinfected with 70% alcohol. |
| - Cleaner cleans isolated areas last, then goes home. |
| **Personal Protective Equipment (PPE)** |
| - To accept client from the hospital there must be sufficient PPE, either provided by hospital or from our own stock. |
| - For new admissions and new clients in home care, employees use PPE the first 7 days |
| - For new applications for terminal overnight care, the organization will accept the client only if supply of PPE is sufficient. |
| - Employees who have been in contact with a confirmed covid patient are requested to wear a surgical mouth-nose mask and gloves, even if they themselves have no symptoms (yet), to protect other clients. |
| - Person who accompanies client for hospital visit is required to wear a face mask. |
| - Instruct personnel about face masks. |
| - Collect face masks and aprons for reuse. |
| - Aprons can be worn for up to one shift, including when the apron keeps the isolation gown visibly clean. If the isolation gown gets wet or dirty during a shift: change. |
| - Staff will always use face masks when a client is quarantined. |
| - Only face mask in case client has symptoms. |
| - Employees wear masks in the covid-cohort and on the covid-unit. |
| - In case of urgent family visits to client, the organization provides PPE to prevent outside infections entering the site. |
| - Employees without symptoms do not wear face masks, that is not necessary. |
| - Due to apron shortage only 3 care moments per day in individual isolation. |
| **Other** |
| - Leave contaminated items in a deceased resident's room for 72 hours. After that, do not move clean items across dirty hallway. |
| - Do not use one lift, only for 'contaminated' items. |
| - No mouth-to-mouth resuscitation, chest compression only. |
| - Clients only go to the hospital for check-up appointments if the specialist feels it is necessary. Then we must facilitate this. Otherwise, appointment by telephone or rescheduled. |
| - Day care: option only for extreme cases, recommendation max. 3 persons. |
| - All care and treatment only if really necessary. Hairdresser/pedicure not yet. Use face masks. |
| - Regular treatments in hospitals are started again. Clients may, only when medically necessary, go to a hospital treatment together with a carer if both are free of symptoms and the carer has not had unprotected contact with a person infected with covid. |
| - To protect resident from outside influences, they cannot go to partner’s funeral 'outside the house', unless quarantine is possible upon return. |
| - Test caregivers with mild symptoms who have client contact. |
| - Expand testing opportunities to all employees who have symptoms. |
