# Supplementary material for: Priority measures to prevent infections and maintain residents’ well-being during COVID-19 outbreaks in nursing homes: Consensus among staff and resident representatives determined in an online nominal group technique study
Source: Int J Nurs Stud Adv. 2023 Jul 13;5:100142. doi: 10.1016/j.ijnsa.2023.100142 (PMC11080460; doi:10.1016/j.ijnsa.2023.100142)
Supplement: Supplementary file 2 [file mmc2.docx]

**Supplement II: Overlap between measures prioritized to prevent infections among residents and measures prioritized to maintain residents’ well-being during COVID-19 outbreaks in nursing homes**

**
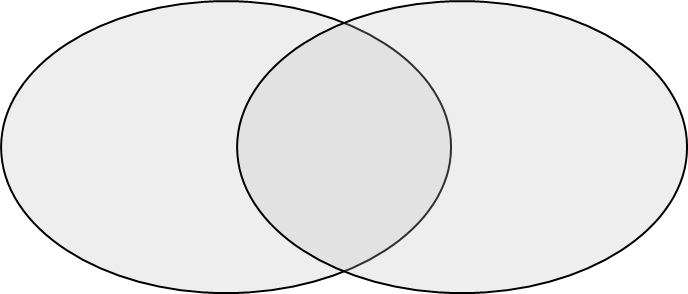
**

- exceptions to visitor bans
- visitor policies
- cohort isolation
- testing and subsequent isolation
- isolation measures
- testing
- use of PPE around (suspected) infected residents
- preparation for outbreaks by COVID-19 outbreak teams

**Infection prevention Well-being maintenance**
