# Supplementary material for: Priority measures to prevent infections and maintain residents’ well-being during COVID-19 outbreaks in nursing homes: Consensus among staff and resident representatives determined in an online nominal group technique study
Source: Int J Nurs Stud Adv. 2023 Jul 13;5:100142. doi: 10.1016/j.ijnsa.2023.100142 (PMC11080460; doi:10.1016/j.ijnsa.2023.100142)
Supplement: Supplementary file 3 [file mmc3.docx]

**Supplement III: quotes from part 2 of the panel conversations regarding decision making**

**Quote 1:** *“the measures taken were rather directive, based on national decisions. [..] The client council was informed of the measures and was not consulted beforehand.”* (resident representative A; August 2020, well-being panel)

**Quote 2:** “*In my opinion, the early communication was more in the form of announcements, not of consultation. Except when the visiting arrangements were expanded. In the beginning, the visitor policies were also just an order from above.”* (manager; July 2020, infection prevention panel)

**Quote 3:** *“If advice needs to be given quickly, it becomes difficult to ask your local client council for their opinion. [..] At that time the client council was not allowed to join the outbreak team. But the discussions often take place ad hoc and then they can’t attend.”* (resident representative B; November 2020, infection prevention panel).

**Quote 4:** *“Nurses are also not very often included in decision-making. Comments don’t make it past location managers who are very busy, and so they don’t end up in the right place.”* (nurse; June 2020, well-being panel)

**Quote 5**: *“In the beginning they [COVID-19 outbreak team] were very strict about those are government rules. [..] It’s different now, but the strictness of that time caused a lot of irritation and complaints.”* (psychologist A; August 2020, well-being panel)

**Quote 6:** *“I get the speed, but I also understand that people feel they were not heard and would have liked to give their opinion. But that, well.. so you only get two days, one day to respond and everyone responds..”* (psychologist B; June 2020,well-being panel)

**Quote 7:** *“First it was all very top-down, now this has changed. We are doing it together and we jointly determine our relationships. The scenarios provide a clear framework, and are known to everyone, client council and works council. Our organization very much wants to develop policy from the work floor.”* (policy advisor; November 2020, infection prevention panel)
